# Isolation and identification of *Pseudoxanthomonas winnipegensis* from blood culture by MALDI-MS

**DOI:** 10.3389/fcimb.2026.1755501

**Published:** 2026-02-16

**Authors:** Satomi Takei, Tatsuya Nagasawa, Yuji Sekiguchi, Junya Fujimura, Kanae Teramoto, Mitsuru Wakita, Teruo Kirikae, Yuki Uehara, Tatsuya Tada, Yoko Tabe

**Affiliations:** 1 Department of Clinical Laboratory Medicine, Juntendo University Graduate School of Medicine, Tokyo, Japan; 2 Department of Clinical Microbiology Analysis Development Research, Juntendo University Graduate School of Medicine, Tokyo, Japan; 3 Department of Clinical Laboratory, Juntendo University Hospital, Tokyo, Japan; 4 Molecular Biosystems Institute, National Institute of Advanced Industrial Science and Technology (AIST), Tsukuba, Ibaraki, Japan; 5 Department of Pediatrics, Juntendo University Graduate School of Medicine, Tokyo, Japan; 6 Analytical & Measurement Instruments Division, Shimadzu Corporation, Kyoto, Japan; 7 Department of Microbiome Research, Juntendo University Graduate School of Medicine, Tokyo, Japan; 8 Department of Microbiology, Juntendo University Graduate School of Medicine, Tokyo, Japan; 9 Department of Clinical Laboratory Technology, Juntendo University Faculty of Medical Science, Chiba, Japan

**Keywords:** blood culture, MALDI-MS, Pseudoxanthomonas winnipegensis, ribosomal protein, whole-genome

## Abstract

*Pseudoxanthomonas* is a genus primarily isolated from environmental samples and causes opportunistic infections. In this study, we conducted a detailed investigation of *P. winnipegensis*, which was isolated for the first time from a blood sample in Japan in 2022, and evaluated five *Pseudoxanthomonas* species, including *P. kaohsiungensis*, *P. japonensis*, *P. mexicana*, *P.* sp*adix* and *P. winnipegensis*, that cause human infections. However, it is difficult to identify accurately by routine microbiological testing. Our analysis revealed that matrix-assisted laser desorption/ionization mass spectrometry (MALDI-MS) classification is feasible when using the mass peaks corresponding to ribosomal proteins L29 and L33, as well as the cold-shock protein CspA, as marker peaks. These findings indicate the potential of MALDI-MS for the rapid and reliable detection of *Pseudoxanthomonas* species in routine microbiological diagnostics.

## 1 Introduction

Members of the genus *Pseudoxanthomonas* were, first described in 2000 by Finkmann et al. and are known to be Gram-negative, aerobic, rod-shaped, motile and oxidase-positive bacteria (Finkmann et al., 2000). To date, the genus *Pseudoxanthomonas* comprises 20 validly published species, which have been isolated from a variety of environmental sources, including soil and water samples^1^.

To our knowledge, only four reports have described *Pseudoxanthomonas* species isolated from human clinical samples. *P. kaohsiungensis* was isolated from a blood culture in Taiwan in 2003 (Kuo and Lee, 2018), *P. japonensis* from blood culture in Sweden in 1987^2^, *P. mexicana* from a urine sample in Germany in 2002 (Thierry et al., 2004), and *P. winnipegensis* from respiratory sample in Canada in 2013 (Bernard et al., 2020).

In clinical laboratories, *Pseudoxanthomonas* species are identified using matrix-assisted laser desorption/ionization mass spectrometry (MALDI-MS), but some species cannot be correctly identified because they are not registered in the database (Wang et al., 2014; Bernard et al., 2020). Although 16S rRNA genes and whole-genomes sequencing can identify *Pseudoxanthomonas* species correctly, they are difficult to use routinely due to its high cost and time-consuming procedures (Cutiño-Jiménez et al., 2020). The susceptibility patterns of *Pseudoxanthomonas* species are unclear, therefore it is important to accumulate surveillance data from clinical laboratories (Thierry et al., 2004; Bernard et al., 2020).

GPMsDB-dbtk is a new software for genomically predicting the theoretical protein mass database for mass spectrometry and MicrobialTrack is a software for identifying bacterial strains by MALDI-MS data (Sekiguchi et al., 2023; Nakagawa et al., 2025). The predicted proteins were experimentally validated to be the correspond proteins (Sun et al., 2006; Teramoto et al., 2019). In this study, we analyzed species identification using MicrobialTrack and evaluated three biomarker peaks, including L29, L33 and CspA, annotated by GPMsDB-dbtk to distinguish five *Pseudoxthanthomonas* species.

In this study, we first isolated *P. winnipegensis* in blood cultures that caused bacteremia. We attempted to identify five *Pseudoxanthomonas* species, including *P. kaohsiungensis*, *P. japonensis*, *P. mexicana*, *P.* sp*adix* and *P. winnipegensis* that cause infections against humans by MALDI-MS peaks.

## 2 Materials and methods

### 2.1 Bacterial strains

*P. winnipegensis* strain JUPW001 was isolated from a blood sample of a 17-year-old woman patient at Juntendo University Hospital, Tokyo, Japan in September 2022. The patient was admitted for suspected central line-associated bloodstream infection due to recurrent fever and elevated C-reactive protein (CRP). Treatment with piperacillin/tazobactam (4.5 g/day) was started and her symptoms improved 2 days after onset. Two sets of blood cultures taken at the time of fever were positive after 36 hours. Three days after treatment, blood cultures were negative. The samples of the positive blood culture were stained and were cultured on AccuRate™ separated Sheep Blood Agar/Chocolate Agar EXII (Shimadzu Diagnostics Co., Kyoto, Japan) and BTB lactose agar (Eiken Chemical Co. Ltd., Tokyo, Japan) at 35°C. The type strain of *P. winnipegensis* NCTC 14396^T^ was obtained from National Collection of Type Cultures (Salisbury, UK). Type strains of *P. kaohsiungensis* CCUG 55854^T^, *P. mexicana* CCUG 49454^T^, *P. japonensis* CCUG 48231^T^, and *P.* sp*adix* CCUG 53828^T^ were obtained from the Culture Collection, University of Göteborg (CCUG), Sweden. All isolates were cultured aerobically at 35°C on 5% sheep blood agar plates (Becton, Dickinson Diagnostic Systems, MD, USA) or Luria-Bertani broth under aerobic conditions at 35 °C.

### 2.2 Whole-genome sequencing

The genomic DNA of the clinical isolate was extracted using DNeasy blood and tissue kits (Qiagen, Tokyo, Japan) and Genomic-tips 20/G (Qiagen). For short-read sequencing, DNA library was prepared using Nextera XT DNA Library Prep Kit (Illumina, San Diego, CA, USA). The genomes were sequenced on the Illumina MiniSeq platform (300 cycles). The raw reads were trimmed and assembled using CLC Genomic Workbench version 10.0.1 (CLC bio, Aarhus, Denmark). For long-read sequencing, DNA library was prepared using Native Barcoding Kit 24 V14 SQK-NBD 114.24 (Oxford Nanopore Technologies, Oxford, UK). Sequencing was performed on R10.4.1 flowcell using MinION Mk1B (Oxford Nanopore Technologies). MinKNOW (version 24.02.8) and Guppy (version 7.3.11) (Oxford Nanopore Technologies) were used for base calling and adapter trimming of raw data. The sequences determined by MiniSeq and MinION were assembled using Unicycler v0.5.0 (Wick et al., 2017). The genome relatedness of the relevant strains was estimated using an average nucleotide identity (ANI) calculator (Yoon et al., 2017) and a Type (Strain) Genome Sever^3^. ANI values were calculated using reference genomes from *P. winnipegensis* (NCTC 14396^T^; genome accession number GCF_004283755). Virulence factors were detected by the virulence factor database (VFDB^4^) and MacSyfinder v2.0 (Abby et al., 2014) for secretion systems (SS).

### 2.3 Phylogenetic analysis

Genome completeness and contamination were assessed using CheckM2 v1.0.1 with lineage_wf and default settings (Parks et al., 2015). A phylogenetic tree was constructed using the kSNP4 software based on pangenome SNPs^5^ (Gardner et al., 2015), and visualized using iTol ver.6^6^. The strains of *P. broegbernensis* (DSM 12573^T^; NZ_JACHGU010000001), *P. daejeonensis* (DSM 17801^T^; NZ_PDWN01000010), *P. dokdonensis* (DSM 21858^T^; NZ_LDJL01000001), *P. gei* (KCTC 32298^T^; NZ_QOVG01000010), *P. helianthin* (NBRC 110414^T^; NZ_JAGKTC010000001), *P. indica* (CCM 7430^T^; NZ_BMCL01000001), *P. japonensis* (CCUG 48231^T^; NZ_PDWW01000010), *P. kalamensis* (DSM 18571^T^; NZ_PDWQ01000001), *P. kaohsiungensis* (CCUG 55854^T^; NZ_PDWO01000010), *P. koreensis* (KCTC 12208^T^; NZ_PDWM01000010), *P. mexicana* (CCUG 49454^T^; NZ_PDWV01000100), *P. putridarboris* (LMG 25968^T^; NZ_JBBWWT010000001), *P. sacheonensis* (DSM 19373^T^; NZ_PDWS01000010), *P. sangjuensis* (DSM 28345^T^; NZ_PDWR01000010), *P.* sp*adix* (CCUG 53828^T^; NZ_RDQN01000001), *P. suwonensis* (DSM 17175^T^; NZ_PDWP01000010), *P. taiwanensis* (DSM 22914^T^; NZ_PDWK01000100), *P. winnipegensis* (NCTC 14396^T^; NZ_SHMH01000001), *P. wuyuanensis* (DSM 100640^T^; NZ_PDWU01000010), *P. yeongjuensis* (DSM 18204^T^; NZ_PDWT01000010), *Stenotrophomonas maltophilia* (NCTC 10257^T^; GCF_900186865.1) were used as reference strains.

### 2.4 Calculation of the theoretical mass of *Pseudoxanthomonas winnipegensis* for MALDI-MS proteotyping

Theoretical masses of proteins encoded in the genomes of *Pseudoxanthomonas* were calculated for the following genomes as part of the development of a genomically predicted protein mass database toolkit (GPMsDB-tk): *P. japonensis* (CCUG 48231^T^; NZ_PDWW01000010), *P. kaohsiungensis* (CCUG 55854^T^; NZ_PDWO01000010), *P. mexicana* (CCUG 49454^T^; NZ_PDWV01000100), *P.* sp*adix* (CCUG 53828^T^; NZ_RDQN01000001), and *P. winnipegensis* (NCTC 14396^T^; NZ_SHMH01000001) (Sekiguchi et al., 2023). The genome sequences were obtained from the NCBI database^7^. The prediction of genes from the genomes obtained in this study was performed using GPMsDB-dbtk v1.0.1^8^ (Sekiguchi et al., 2023).

### 2.5 Bacterial sample preparation for MALDI-MS

Alpha-cyano-4-hydroxycinnamic acid (CHCA) was used as a matrix. To prepare this matrix solution, 10 mg of 4-CHCA was dissolved in 1 mL of solvent consisting of 1% (v/v) trifluoroacetic acid, 35% (v/v) ethanol, 15% (v/v) acetonitrile, and milliQ water. A full loop of bacterial cells was dispersed in 200 μL of distilled water in a microtube and mixed with 800 μL of ethanol. The suspensions were briefly vortexed and centrifuged at 15, 000 g for 2 min. The pellets were then dried for 5 min. The pellets were suspended in 50 μL of 70% formic acid, vortexed, suspended in 50 μL of acetonitrile, and centrifuged at 15, 000 g for 2 min. Supernatants were analyzed by MALDI-MS according to the manufacturer’s instruction.

### 2.6 MALDI-MS measurement

MALDI-MS measurements were performed in positive linear mode using MALDI-8020 RUO (Shimadzu Corporation, Kyoto, Japan) and Microflex LT/SH (Bruker Daltonics, Germany) equipped with a 200 Hz Nd: YAG laser (355 nm) and 60 Hz nitrogen laser (337 nm), respectively. Before sample analysis, the MALDI-MS instrument was mass-calibrated externally using six peaks with *m/z* 4365.4, 5381.4, 6411.6, 7274.0, 8369.8, and 10300.1 from *Escherichia coli* DH5α. More than five individual mass spectra were acquired for each bacterial extract in the range of *m/z* 2, 000-20, 000. The assignment of the peak was performed using eMSTAT Solution™ software (Shimadzu Corporation). Species identifications were performed by the MicrobialTrack software v1.1.0 (Shimadzu Corporation) (Sekiguchi et al., 2023; Nakagawa et al., 2025) and MBT Compass 4.1 with Microflex LT/SH (Bruker Daltonics).

### 2.7 Cluster analysis for *Pseudoxanthomonas* type strains using L29, L33 and CspA

For biomarker validation, the theoretical mass of L29, L33 and CspA was calculated for 15 *Pseudoxanthomonas* type strains: *P. broegbernensis* (DSM 12573^T^; NZ_JACHGU010000001), *P. daejeonensis* (DSM 17801^T^; NZ_PDWN01000010), *P. dokdonensis* (DSM 21858^T^; NZ_LDJL01000001), *P. gei* (KCTC 32298^T^; NZ_QOVG01000010), *P. helianthin* (NBRC 110414^T^; NZ_JAGKTC010000001), *P. indica* (CCM 7430^T^; NZ_BMCL01000001), *P. kalamensis* (DSM 18571^T^; NZ_PDWQ01000001), *P. koreensis* (KCTC 12208^T^; NZ_PDWM01000010), *P. putridarboris* (LMG 25968^T^; NZ_JBBWWT010000001), *P. sacheonensis* (DSM 19373^T^; NZ_PDWS01000010), *P. sangjuensis* (DSM 28345^T^; NZ_PDWR01000010), *P. suwonensis* (DSM 17175^T^; NZ_PDWP01000010), *P. taiwanensis* (DSM 22914^T^; NZ_PDWK01000100), *P. wuyuanensis* (DSM 100640^T^; NZ_PDWU01000010), and *P. yeongjuensis* (DSM 18204^T^; NZ_PDWT01000010). A phylogenetic tree was constructed using SRplot with the unweighted pair group method with arithmetic mean (UPGMA) (Tang et al., 2023).

### 2.8 Accession numbers

The whole-genome sequence of *P. winnipegensis* JUPW001 obtained in this study has been deposited in GenBank under the accession number AP044739^9^.

## 3 Results

### 3.1 Identification of *P. winnipegensis* JUPW001

Samples of the positive blood culture were stained as Gram-negative rod. After 24 h of incubation, a large number of oxidase-positive and yellow-colored colonies were observed on the agars. The isolate was identified as “*Pseudoxanthomonas* species” using MALDI-MS with MBT Compass 4.1 by Microflex LT/SH (Bruker Daltonics).

The ANI value using whole-genome sequence of the isolate was 97.4% identical to the sequence of *P. winnipegensis* (NCTC 14396^T^; NZ_SHMH01000001). Therefore, the isolate was confirmed to be *P. winnipegensis* and designated as *P. winnipegensis* JUPW001.

*P. winnipegensis* JUPW001 had flagellum, type 1 secretion system (T1SS), T2SS, T4SS, T5SS and type IVa pilus (T4aP). In particular, T4SS included VirB2 to VirB6 and VirB8 to VirB11.

### 3.2 Phylogenic analysis

**Figure 1:**
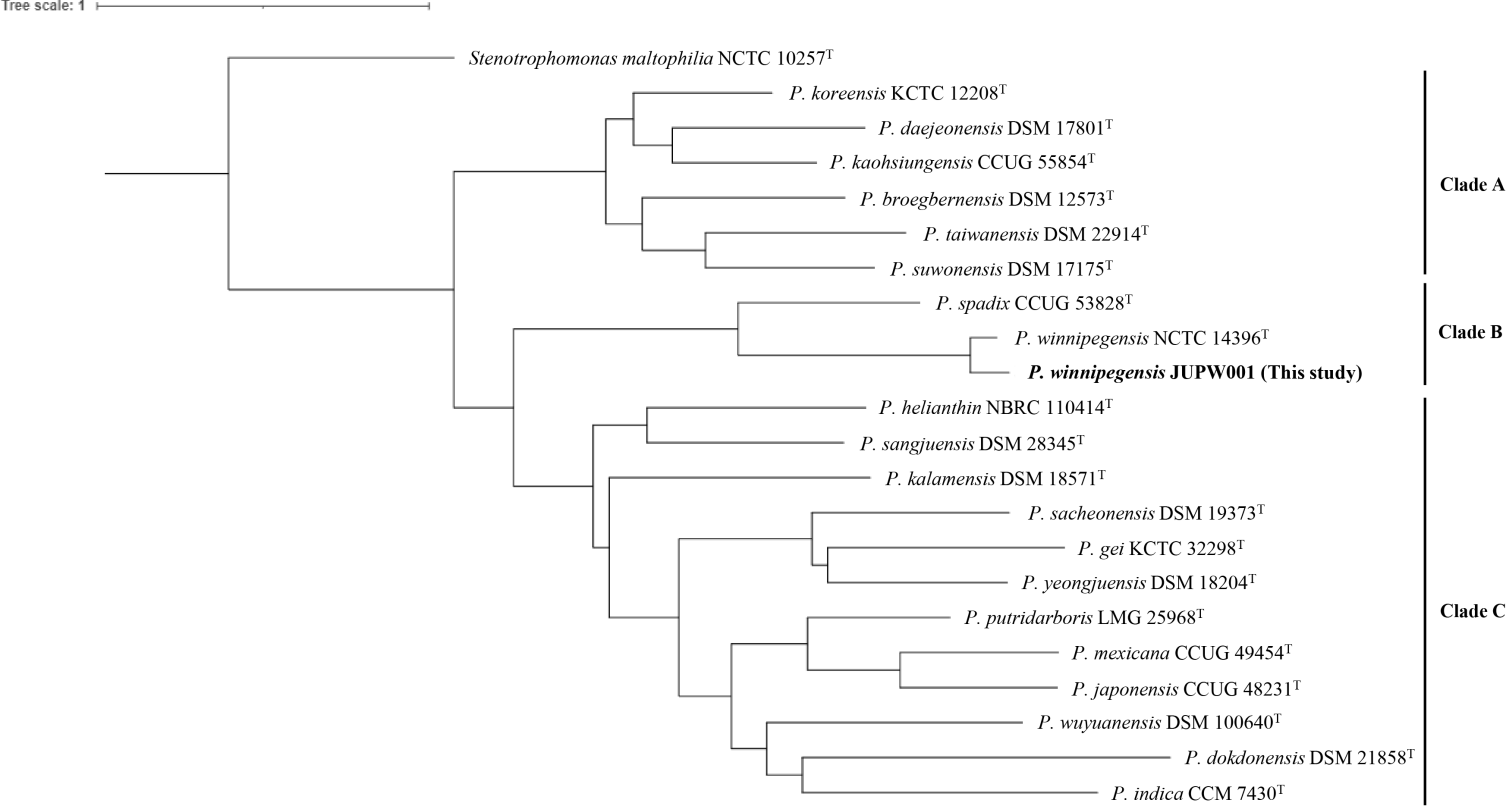
Phylogenetic tree of one clinical strain and 20 type strains of *Pseudoxanthomonas* species. Phylogenetic trees were constructed using kSNP4 software based on pangenome SNPs (footnote 5) (Gardner et al., 2015) and visualized using iTol ver.6 (footnote 6).

As shown in **Figure 1**, the phylogeny was separated by the genus level between *Pseudoxanthomonas* and *Stenotrophomonas*. The phylogenetic tree revealed three clades: A, B, and C. *P. winnipegensis* JUPW001 belonged to clade B and the species was close to *P.* sp*adix* (**Figure 1**). In clade A, *P. kaohsiungensis* (GenBank accession no. NZ_PDWO01000010) was isolated from humans (Kuo and Lee, 2018). Other previously reported clinical isolates, *P. japonensis* (accession no. NZ_PDWW01000010) and *P. mexicana* (accession no. NZ_PDWV01000100), belonged to clade C (Thierry et al., 2004). The other species were mainly isolated from soil or water environments.

### 3.3 MALDI-MS analysis of *Pseudoxanthomonas* species

The bacterial identification for *Pseudoxanthomas* species was performed using MicrobialTrack, which is a software for peak matching by predicted mass values using the annotated protein sequences. The MicrobialTrack analysis revealed that *P. winnipegensis* JUPW001, *P. winnipegensis* NCTC 14396^T^, *P.* sp*adix* CCUG 53828^T^, *P. japonensis* CCUG 48231^T^, *P. mexicana* CCUG 49454^T^, and *P. kaohsiungensis* CCUG 55854^T^ were correctly identified with high reliability (**Table 1**). In this analysis, the number of ribosomal proteins peaks in each strain were from 19 to 28 and annotated proteins except for ribosomal proteins were from 8 to 19. In contrast, Microflex LT/SH analysis using the Biotyper database correctly identified *P. kaohsiungensis*, *P. mexicana*, and *P.* sp*adix* at the species level, whereas *P. japonensis* was misidentified and *P. winnipegensis* was classified only at the genus level (**Table 1**).

**Table 1:** The analysis using MicrobialTrack of *Pseudoxanthomonas* species.

Strains	MicrobialTrack			MALDI biotyper
	Closest species (Reliability)	Annotated ribosomal proteins	Annotated proteins except for ribosomal proteins	Closest species (score values)
*P. winnipegensis* JUPW001	*P. winnipegensis* (Very High)	24	11	*Pseudoxanthomonas* species (2.19)
*P. winnipegensis* NCTC 14396^T^	*P. winnipegensis* (Very High)	21	17	*Pseudoxanthomonas* species (2.06)
*P.* sp*adix* CCUG 53828^T^	*P.* sp*adix* (Very High)	19	8	*P.* sp*adix* (2.40)
*P. japonensis* CCUG 48231^T^	*P. japonensis* (Very High)	24	17	*P. mexicana* (1.77)
*P. mexicana* CCUG 49454^T^	*P. mexicana* (Very High)	23	19	*P. mexicana* (2.38)
*P. kaohsiungensis* CCUG 55854^T^	*P. kaohsiungensis* (Very High)	28	19	*P. kaohsiungensis* (2.65)

NA, not assigned.

### 3.4 Biomarker proteins to distinguish *Pseudoxanthomonas* species

**Figure 2:**
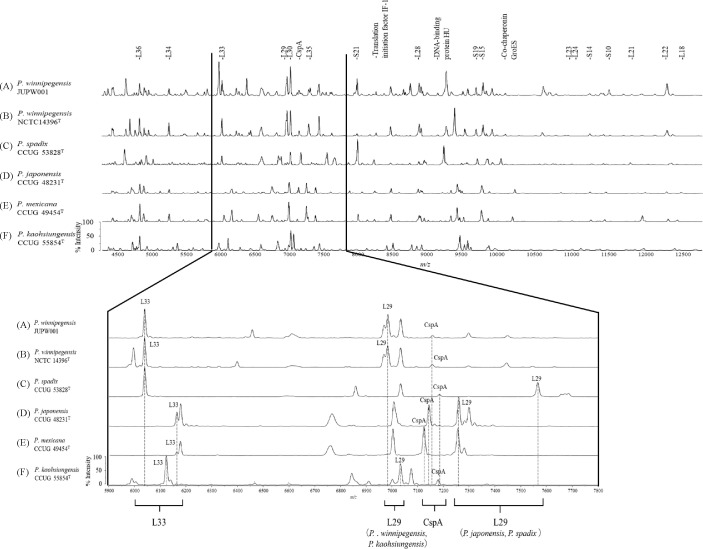
Representative mass spectra of *Pseudoxanthomonas* species, including *P. winnipegensis* JUPW001 **(A)**, *P. winnipegensis* NCTC 14396^T^**(B)**, *P.* sp*adix* CCUG 53828^T^**(C)**, *P. japonensis* CCUG 48231^T^**(D)**, *P. mexicana* CCUG 49454^T^**(E)**, and *P. kaohsiungensis* CCUG 55854^T^**(F)**. Upper figure indicates mass spectra from *m/z* 4, 500 to 12, 500. The annotated peaks indicate the assigned peaks based on the calculated masses within the tolerance at 500 ppm. Lower figure indicates amplifications of the *m/z* 5, 900 to 7, 800 section of mass spectra and variations of *m/z* values for peaks ribosomal L29, L33 and CspA.

The candidate mass peaks of MALDI-MS analysis for the five type strains, including *P. kaohsiungensis*, *P. japonensis*, *P. mexicana*, *P.* sp*adix* and *P. winnipegensis*, are shown in **Figure 2**. In the MALDI-MS profiles, 21 major mass peaks were commonly detected and successfully annotated with predicted protein names in *P. winnipegensis* JUPW001 and NCTC 14396^T^ (**Table 2**). Of these 21 annotated peaks, 17 were predicted as ribosomal subunit proteins, and the remaining four were co-chaperonin GroES, cold shock-like protein (CspA), DNA-binding protein HU and translation initiation factor IF-1. As shown in **Table 2**, compared to the theoretical mass peaks of *P. winnipegensis* NCTC 14396^T^, most of the peaks were different from the theoretical mass peaks of *P.* sp*adix* CCUG 53828^T^, *P. japonensis* CCUG 48231^T^, *P. mexicana* CCUG 49454^T^, and *P. kaohsiungensis* CCUG 55854^T^. To distinguish five *Pseudoxanthomonas* at the species level using MALDI-MS, the combination of appropriate peaks of ribosomal L29, L33 and CspA were selected as biomarkers.

**Table 2:** Annotated peaks of clinical and type strains of *Pseudoxanthomonas* species.

Biomarker protein	*P. winnipegensis* JUPW001				*P. winnipegensis* NCTC 14396^T^				*P. spadix* CCUG 53828^T^				*P. japonensis* CCUG 48231^T^				*P. mexicana* CCUG 49454^T^				*P. kaohsiungensis* CCUG 55854^T^			
	Calculated masses (*m/z*)	Average	SE	Peak numbers (n=5)	Calculated masses (*m/z*)	Average	SE	Peak numbers (n=5)	Calculated masses (*m/z*)	Average	SE	Peak numbers (n=5)	Calculated masses (*m/z*)	Average	SE	Peak numbers (n=5)	Calculated masses (*m/z*)	Average	SE	Peak numbers (n=5)	Calculated masses (*m/z*)	Average	SE	Peak numbers (n=5)
L36	4842.9	4842.9	0	5	4842.9	4842.7	0.16	5	4842.9	4842.9	0	5	4842.9	4842.9	0	5	4842.9	4842.9	0	5	4842.9	4842.9	0	5
L34	5271.2	5270.4	0.23	5	5271.2	5271.4	0.09	5	5271.2	5270.1	0.48	5	5267.2	5267.4	0.05	5	5267.2	5267.3	0.03	5	5324.3	5324.6	0.11	5
L33	6039.0	6039.9	0.31	5	6039.0	6040.6	0.16	5	6039.0	6039.0	0.10	5	6163.2	6163.5	0.06	5	6163.2	6163.2	0.06	5	6122.2	6123.6	0.09	5
L32	6968.8	NA	NA	0	6968.8	NA	NA	0	6857.6	6859.2	0.35	5	7014.8	NA	NA	0	7002.8	7004.2	0.06	5	7000.8	7001.5	0.15	5
L29	6983.1	6980.0	0.48	5	6983.1	6983.6	0.16	5	7565.7	7565.3	0.07	5	7257.5	7257.9	0.09	5	7296.6	NA	NA	0	7032.1	7032.5	0.15	5
L30	7033.2	7033.6	0.21	5	7033.2	7033.3	0.18	5	7033.2	7032.8	0.11	5	7005.2	7005.9	0.14	5	7005.2	NA	NA	0	7074.2	7074.7	0.18	5
CspA	7155.9	7159.0	0.10	5	7155.9	7157.9	0.54	5	7183.9	7185.1	0.12	5	7141.9	7142.2	0.12	5	7125.9	7126.1	0.07	5	7178.9	7179.8	0.27	5
L35	7296.6	7296.3	0.12	5	7296.6	7297.5	0.24	5	7296.6	7296.3	0.14	5	7296.6	7297.6	0.07	5	7282.6	7282.2	0.05	5	7368.7	7369.5	0.17	5
Translation initiation factor IF-1	8273.6	8273.8	0.19	5	8273.6	8273.2	0.16	5	8245.6	8244.5	0.14	5	8245.6	8245.6	0.14	5	8245.6	8244.7	0.12	5	8285.6	8285.6	0.06	5
S21	7997.8	7996.2	0.20	5	7997.8	7997.8	0.12	5	8483.8	8484.2	0.25	5	8483.8	8484.1	0.13	5	8483.8	8483.1	0.08	5	8423.3	8422.9	0.05	5
L28	8897.2	8897.0	0.24	5	8897.2	8897.2	0.00	5	8897.2	8897.2	0.00	5	8878.2	8878.2	0.00	5	8895.3	8894.9	0.38	5	8785.0	8785.0	0.00	5
L27	8898.1	NA	NA	0	8898.1	NA	NA	0	8997.3	NA	NA	0	8937.3	8938.0	0.27	5	8967.3	8967.1	0.08	5	8858.1	8858.0	0.07	5
DNA-binding protein HU	9289.6	9292.7	0.33	5	9289.6	9289.8	0.05	5	9259.5	9260.0	0.15	5	9444.9	9445.7	0.17	5	9444.9	9444.1	0.06	5	9484.0	9484.6	0.09	5
S19	9823.5	9826.4	0.24	5	9823.5	9824.0	0.07	5	9895.6	9895.2	0.24	5	9795.5	9797.6	0.17	5	9795.5	9796.3	0.14	5	9595.3	9596.7	0.02	5
S15	9954.5	9955.6	0.24	5	9954.5	9954.0	0.13	5	9879.3	NA	NA	0	9813.2	NA	NA	0	9811.2	NA	NA	0	9902.3	9903.0	0.04	5
Co-chaperonin GroES	10146.7	10148.8	0.54	5	10146.7	10146.5	0.05	5	10085.7	10086.8	0.19	5	10276.8	10278.4	0.16	5	10247.8	10247.8	0.19	5	10204.7	10205.7	0.18	5
L23	11014.5	11016.4	0.17	5	11014.5	11012.2	0.54	4	11002.4	11002.9	NA	1	11111.7	NA	NA	0	11111.7	11113.0	0.42	5	10879.4	10879.0	0.40	2
L24	11059.6	11059.8	0.30	5	11059.6	NA	NA	0	11113.7	11114.1	0.28	5	11073.6	NA	NA	0	11115.7	NA	NA	0	11113.7	11113.9	0.20	5
S14	11372.2	11373.1	0.35	5	11372.2	11370.8	0.24	5	11400.2	11400.4	0.44	5	11368.2	11370.1	0.32	5	11386.2	11385.1	0.42	5	11235.0	11235.6	0.20	5
S10	11572.3	11572.3	0.29	5	11572.3	11570.6	0.18	4	11572.3	11572.1	0.43	5	11600.4	11603.4	0.40	5	11584.4	11585.6	0.35	5	11512.3	11512.7	0.22	5
L21	11860.7	11862.9	0.36	5	11860.7	11860.2	0.28	5	11870.7	11872.0	NA	1	10574.4	NA	NA	0	11475.3	11475.8	0.72	5	11314.1	11315.1	0.23	5
L22	12317.4	12318.4	0.36	5	12317.4	12316.2	0.19	5	12224.3	12224.3	NA	1	12172.4	12171.1	0.60	2	12172.5	NA	NA	0	12018.2	12019.1	0.31	5
L18	12572.4	12574.8	0.46	5	12572.4	12572.1	0.25	5	12641.5	12641.6	1.48	3	12687.5	12690.0	0.53	5	12641.5	12637.8	0.54	5	12596.5	12596.5	0.39	5

CspA; Cold shock-like protein, *m/z*, mass to charge ratio; NA, not assigned; SE, standard error.

As shown in **Figure 2** and **Table 2**, the corresponding theoretical peaks of ribosomal protein L29 were *m/z* 6983.1 for *P. winnipegensis* NCTC 14396^T^; *m/z* 7565.7 for *P.* sp*adix* CCUG 48231^T^; *m/z* 7257.5 for *P. japonensis* CCUG 48231^T^ and *m/z* 7032.1 for *P. kaohsiungensis* CCUG 55854^T^ (**Figure 2**; **Table 2**). The peak at *m/z* 7296.6 for L29 of *P. mexicana* CCUG 49454^T^ were not detected, although the theoretical peaks were calculated by GPMsDB-dbtk. The corresponding theoretical peaks of the ribosomal protein L33 were *m/z* 6039.0 for *P. winnipegensis* NCTC 14396^T^ and *P.* sp*adix* CCUG 48231^T^; *m/z* 6163.2 for *P. japonensis* CCUG 48231^T^ and *P. mexicana* CCUG 49454^T^; *m/z* 6122.2 for *P. kaohsiungensis* CCUG 55854^T^ (**Figure 2**; **Table 2**). The corresponding theoretical peaks of CspA were *m/z* 7155.9 for *P. winnipegensis* NCTC 14396^T^; *m/z* 7183.9 for *P.* sp*adix* CCUG 48231^T^; *m/z* 7141.9 for *P. japonensis* CCUG 48231^T^; *m/z* 7125.9 for *P. mexicana* CCUG 49454^T^ and *m/z* 7178.9 for *P. kaohsiungensis* CCUG 55854^T^ (**Figure 2**; **Table 2**).

**Figure 3:**
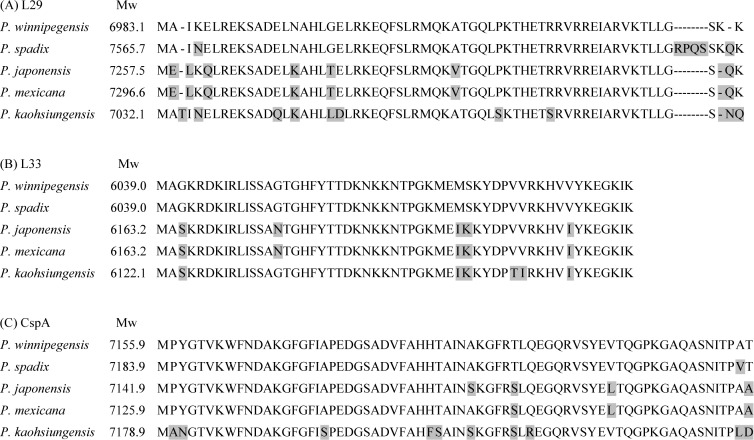
Alignment of amino acid sequences of the ribosomal protein L29 **(A)**, L33 **(B)** and CspA **(C)** of *Pseudoxanthomonas* species. Amino acid substitutions are shaded in gray.

Compared to the amino acid sequence of L29 in *P. winnipegensis*, there were 6 amino acid substitutions in *P.* sp*adix*, 8 in *P. japonensis*, 8 in *P. mexicana* and 11 in *P. kaohsiungensis* (**Figure 3A**). Compared to the amino acid sequence of L33 in *P. winnipegensis*, there were 5 substitutions in *P. japonensis*, 5 in *P. mexicana*, and 6 in *P. kaohsiungensis* (**Figure 3B**). Compared to the amino acid sequence of CspA of *P. winnipegensis*, there were 1 substitution in *P.* sp*adix*, 4 in *P. japonensis*, 3 in *P. mexicana*, and 10 in *P. kaohsiungensis* (**Figure 3C**).

MALDI-MS proteotyping was able to distinguish *P. winnipegensis*, *P.* sp*adix*, *P. japonensis*, *P. mexicana* and *P. kaohsiungensis* using the three biomarkers, including L29, L33 and CspA.

### 3.5 Cluster analysis for 20 *Pseudoxanthomonas* type strains

Cluster analysis using L29, L33 and CspA revealed that 20 *Pseudoxanthomonas* type strains were distinguished by the three theoretical mass peaks (**Supplementary Table S1** and **Supplementary Figure S1**). The close cluster such as *P. japonensis, P. putridarboris* and *P. wuyuanensis* were theoretically separated by L29 or CspA, whereas *P. sacheonensis* and *P. yeongjuensis* were theoretically separated by CspA (**Supplementary Figure S1**).

## 4 Discussion

*Pseudoxanthomonas* is a relatively recently characterized genus and reports of human infection remain rare. Accurate and rapid diagnosis of *Pseudoxanthomonas* infections in immunocompromised patients is critical, because it is known that the species have several virulence factors, including T1SS, T2SS, T3SS, T4SS and T4aP. Especially, T4SS plays crucial roles in pathogens in the delivery of effector proteins (Green and Mecsas, 2016; Costa et al., 2024). The T4SS signature protein, VirB4, in *Xanthomonadaceae* is known to kill other bacterial cells and have advantage to competitive growth in mixed bacterial communities (Souza et al., 2015). VirB4 is also known the only ubiquitous protein with recognizable homologs in all known T4SS (Guglielmini et al., 2014). The T4SS effector protein, VirB10, in *S. maltophilia* is known to reduce apoptotic activity and promote its growth (Nas et al., 2019). *P. winnipegensis* JUPW001 harbored both of VirB4 and VirB10 protein.

**Figure 4:**
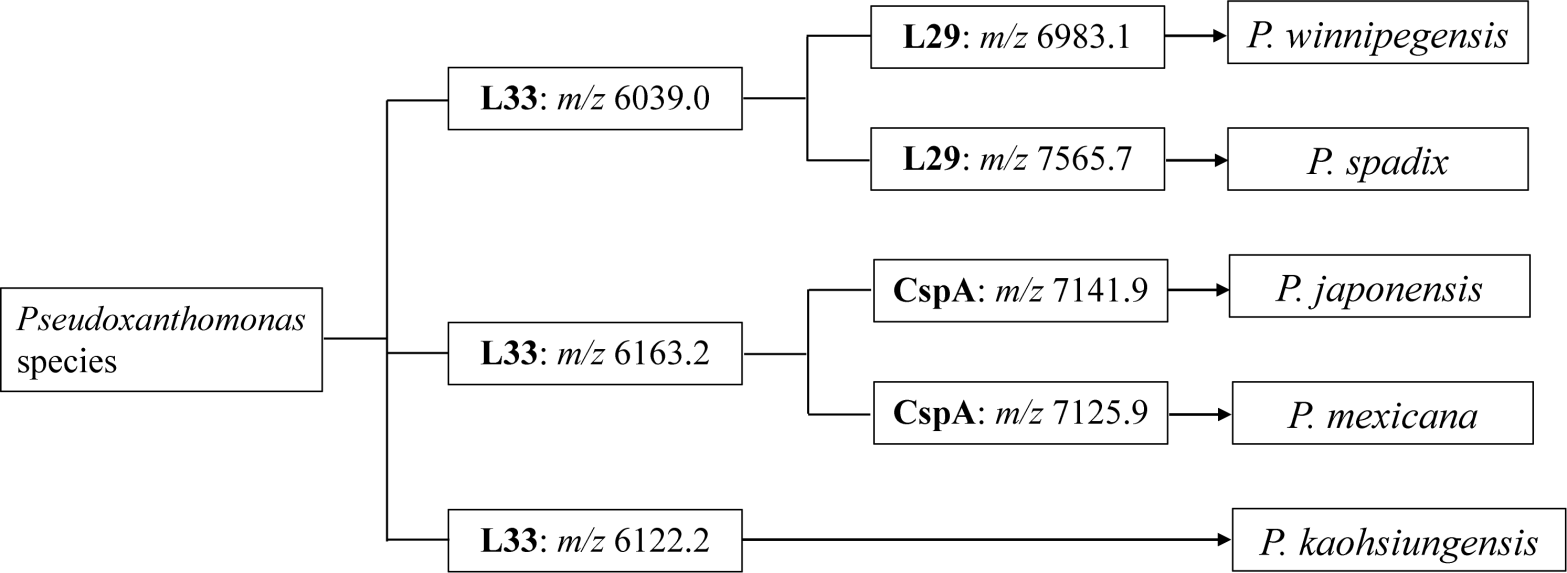
Workflow for the identification of phylotypes of five *Pseudoxanthomonas* species by MALDI-MS proteotyping.

The biomarker peaks of L29, L33 and CspA are useful for distinguishing *Pseudoxanthomonas* at the species level. Our study suggests that at least five *Pseudoxanthomonas* species can be separated using MALDI-MS, even if MicrobialTrack is not installed. **Figure 4** shows the workflow for the rapid identification of *Pseudoxanthomonas* species by MALDI-MS, describing L33 for three groups: *P. winnipegensis/P.* sp*adix*, *P. japonensis/P. mexicana* and *P. kaohsiungensis*, L29 for *P. winnipegensis* and *P.* sp*adix*, and CspA for *P. japonensis* and *P. mexicana*. Analysis using the theoretical masses of the other 15 *Pseudoxanthomonas* species revealed that it is possible to classify 20 *Pseudoxanthomonas* species, including *P. kaohsiungensis*, *P. japonensis*, *P. mexicana*, *P.* sp*adix* and *P. winnipegensis*, using the three biomarker peaks (**Supplementary Table S1** and **Supplementary Figure S1**).

The identification of MALDI-MS proteotyping using the MicrobialTrack will be useful for species lacking mass spectral reference libraries such as *Pseudoxanthomonas* species. Previous study on the *P. winnipegensis* identification using MALDI-MS have shown that the misidentification as *P.* sp*adix* with low scores ranging from 1.22 to 1.38 in Biotyper analysis (Bernard et al., 2020). Most of *Pseudoxanthomonas* species can identify at the genus-level, and only three species, including *P. indica*, *P. mexicana* and *P.* sp*adix*, can identify at the species-level using MALDI reference libraries of MBT Compass 4.1 installed in Biotyper (**Table 1**). It is possible to identify species-level of *Pseudoxanthomonas* using MicrobialTrack if the exact genome sequences and the enough quality of the measured MALDI spectra are obtained from tested bacteria.

This study has a few limitations: first, only one clinical strain was analyzed obtained in this study, and five of 20 species were tested using MALDI-MS. More data on clinical isolates of *Pseudoxanthomonas* species should be collected and the prospective validations are important.

In conclusion, this study reports the first case of human infection caused by *P. winnipegensis* and highlights the potential utility of MALDI-MS proteotyping for rapid and accurate species-level identification of *Pseudoxanthomonas* clinical isolates.

## Data Availability

The datasets presented in this study can be found in online repositories. The names of the repository/repositories and accession number(s) can be found below: https://www.ncbi.nlm.nih.gov/, AP044739.

## Footnotes

1
https://lpsn.dsmz.de/genus/pseudoxanthomonas

2
https://bacdive.dsmz.de/strain/143534

3
https://tygs.dsmz.de

4
http://www.mgc.ac.cn/VFs/

5
https://sourceforge.net/projects/ksnp/

6
https://itol.embl.de/

7
https://www.ncbi.nlm.nih.gov/

8
https://github.com/ysekig/GPMsDB-dbtk

9
http://getentry.ddbj.nig.ac.jp/
