# Supplementary material for: Isolation and identification of Pseudoxanthomonas winnipegensis from blood culture by MALDI-MS
Source: Front Cell Infect Microbiol. 2026 Feb 16;16:1755501. doi: 10.3389/fcimb.2026.1755501 (PMC12950775; doi:10.3389/fcimb.2026.1755501)
Supplement: Supplementary file 1 [file DataSheet1.pdf]

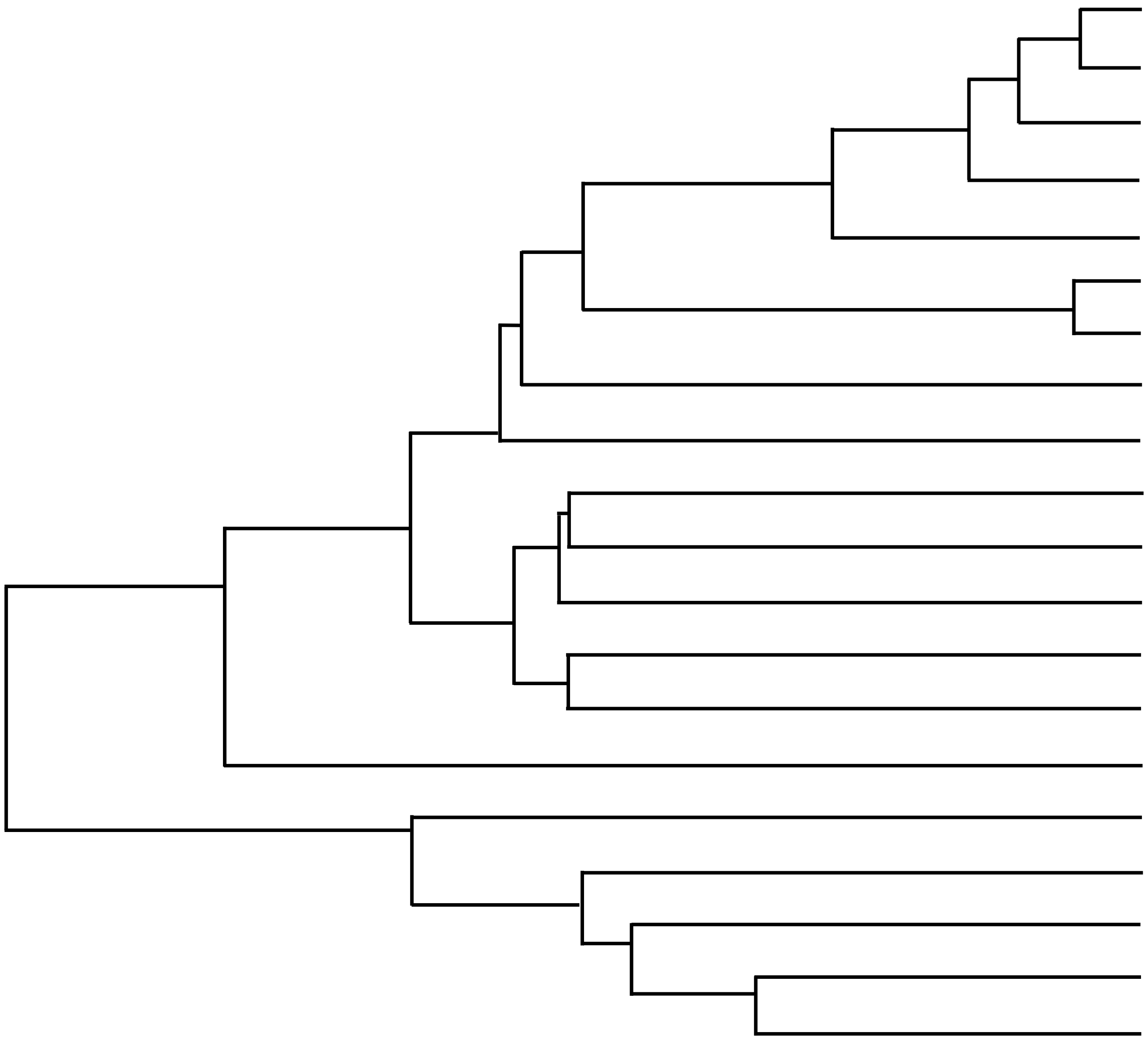

	L33	L29	CspA
P. wuyuanensis DSM 100640^{T}	6163.2	7243.4	7127.8
P. putridarboris LMG 25968^{T}	6163.2	7243.5	7141.9
P. japonensis CCUG 48231^{T}	6163.2	7257.5	7141.9
P. indica CCM 7430^{T}	6163.2	7222.5	6993.7
P. mexicana CCUG 49454^{T}	6163.2	7296.6	7125.9
P. taiwanensis DSM 22914^{T}	6163.2	7142.3	7196.0
P. suwonensis DSM 17175^{T}	6163.2	7144.3	7162.9
P. kalamensis DSM 18571^{T}	6152.2	7041.1	7240.0
P. dokdonensis DSM 21858^{T}	6177.2	7214.5	7072.7
P. sangjuensis DSM 28345^{T}	6138.2	7209.3	7149.9
P. daejeonensis DSM 17801^{T}	6136.2	7116.1	7079.8
P. broegbernensis DSM 12573^{T}	6133.1	7130.2	7150.9
P. helianthin NBRC 110414^{T}	6124.2	9962.4	7151.9
P. kaohsiungensis CCUG 55854^{T}	6122.2	7032.1	7178.9
P. koreensis KCTC 12208^{T}	6220.3	7101.1	7178.9
P. gei KCTC 32298^{T}	6071.1	7693.9	7242.9
P. winnipegensis NCTC 14396^{T}	6039.0	6983.1	7155.9
P. spadix CCUG 53828^{T}	6039.0	7565.7	7183.9
P. yeongjuensis DSM 18204^{T}	6039.0	7286.5	7121.8
P. sacheonensis DSM 19373^{T}	6039.0	7286.5	7155.8

Supplementary Figure 1: Cluster analysis of 20 strains of *Pseudoxanthomonas* species based on theoretical biomarker masses. The tree was constructed using the unweighted pair group method with arithmetic mean (UPGMA) using Srplot.
